# Pathological and Practical Researches on Uterine Inflammation in Puerperal Women

**Published:** 1831

**Authors:** 


**Pathological and Practical Researches on Uterine Inflammation in Puerperal Women.** By *Robert Lee*, M.D. F.R.S. Physician to the British Lying-in Hospital, &c. &c. &c.

[Med. Chir. Transactions, Vol. XVI.]

In the 12th volume (No. XXIV.) of this Journal, page 376—384, we gave an ample analysis of Dr. Lee's paper on Uterine Phlebitis and Phlegmatia Dolens, published in the 15th volume of the Medico-Chirurgical Transactions. The present paper, occupying upwards of 80 pages of the volume in question, deserves undivided attention from the profession, as the result of great and unwearied labour, indefatigable zeal, and talent of a high order. Indeed we cannot help taking this opportunity of awarding our meed of praise to the merits of a young physician, who, if we are not much mistaken, will, ere long, make a distinguished figure in his profession. We do not know, at this moment, a man who better deserves reputation with its accompaniments, than the author of the paper now before us, and we have no doubt that the institution to which he belongs, and the school where he teaches, will give him ample scope for the exertion of those abilities, and the dissemination of that pathological and practical knowledge which he has so zealously and so early acquired. But we must now proceed to an analysis of this important paper.

The febrile and inflammatory diseases of parturient women were long thought to depend on inflammation of the uterus, excited by suppression of the lochial discharge, or on some diseased condition of the animal fluids resulting from pregnancy. In modern times, the greatest diversity of opinion has prevailed respecting the nature and treatment of these acute disorders, some referring them wholly to peritoneal inflammation, while others, overlooking the local affections of the uterine organs, have described them as specific diseases, under the terms puerperal, peritoneal, or child-bed fever.

In the last paper which our author published on Uterine Phlebitis, he ventured to infer that inflammation of the uterine veins is of far more frequent occurrence than has yet been suspected, and that to it must be referred many of those fatal puerperal disorders which have usually been comprehended under the vague designation of puerperal fever or peritonitis.

"At that period I had also arrived at the following conclusion, which subsequent experience has fully confirmed, 'that inflammation of the uterus and its appendages must be considered as essentially the cause of all the destructive febrile affections which follow parturition, and that the various forms they assume, inflammatory, congestive, or typhoid, will, in a great measure, be found to depend on the serous, muscular, or venous tissue of the organ having become affected.'*

From the 1st of January 1827, to the 1st of March 1831, including a period of more than four years, one hundred and twelve cases of well-marked uterine inflammation, have come under my observation in the British Lying-in Hospital, and in public and private practice in the western districts of this metropolis. I have watched the symptoms and progress of these cases with the closest attention, observed the effects of remedies, and where death has taken place, I have carefully examined the alterations of structure, which have remained in the uterine and other organs.

Of forty cases which have proved fatal, the bodies of thirty-four have been examined, and in all of these, which had presented during life, the characteristic symptoms of what has been usually denominated Puerperal Fever, there existed some morbid change from inflammation either in the peritoneal coat of the uterus, or of the uterine appendages, in the muscular tissue, the veins or absorbents of the uterus, to account in a complete and most satisfactory manner for all the constitutional disturbance which had been observed. The peritoneum and uterine appendages were found inflamed in twenty-six cases, in fourteen there existed uterine phlebitis, in eight, inflammation and softening of the muscular tissue of the organ, and in four, the absorbents were distended with pus. The results of these observations, as far as they go, are therefore decidedly opposed to the opinion now generally prevalent in this country, that there is a specific fever, which attacks puerperal women, and which may arise independent of any local affection in the uterine organs, and prove fatal frequently, without leaving any perceptible change in the organization of their different textures." 380.

In the present communication Dr. Lee proposes succinctly to describe the various changes produced by inflammation in the uterine organs subsequent to parturition—to point out the local and constitutional symptoms by which these morbid conditions are characterized during life, and to which combination of symptoms the terms puerperal, peritoneal, or child-bed fever, have been applied by different authors—and, lastly, to describe the treat-

* Med. Chir. Transactions, Vol. XV. p. 465.

ment which experience has led him to consider as the most safe and efficacious.

"The following are the principal modifications of inflammation of the uterus in puerperal women which I have observed.

1st. Inflammation of the peritoneal covering of the uterus, and of the general peritoneal sac.

2dly. Inflammation of the uterine appendages; ovaria, Fallopian tubes, and broad ligaments.

3dly. Inflammation of the muscular or proper tissue of the uterus.

4thly. Inflammation and suppuration of the veins, and absorbent vessels of these organs.

These varieties of uterine inflammation may occur wholly independent of each other, though they are most frequently met with in combination. Peritonitis seldom occurs, without some degree of inflammation of the uterine appendages, but both these textures I have found severely affected, when the muscular coat of the uterus and the veins have been wholly exempt from disease. The venous and muscular tissues of the uterus are also liable to severe attacks of inflammation, without any corresponding affection of the peritoneum, by which they are covered, though it most frequently happens that inflammation when set up, either in the veins or muscular coat, involves also the peritoneum. In the organs of respiration similar varieties of inflammation may be observed, and the pleura, pulmonary texture, and the mucous membrane lining the air passages, may all be separately or simultaneously involved in the same attack. A similar observation may be extended to the brain and its membranes, and to the whole of the digestive organs, and the symptoms which characterize the inflammation of the different tissues, of which these organs are composed, have been as accurately determined, as they possibly can be, in the present state of pathological science."

### I. Inflammation of the Peritoneal Covering of the Uterus, and of the General Peritoneal Sac.

The effects produced by inflammation of the uterine peritoneal coat, in puerperal women, do not differ essentially from those produced by ordinary peritonitis in the male sex. Where inflamed, the membrane becomes vascular, red, apparently thickened, while a secretion or substance of a yellow colour, in the form of a false membrane, is thrown out, producing adhesion of the abdominal viscera to each other. Or a morbid serum, mixed with shreds of albumen or pus, is effused, more or less, into the cavity of the peritoneum.

"Puerperal peritonitis usually commences in the peritoneum of the uterus, and extends from thence with greater or less rapidity, according to the severity of the attack, to the general peritoneal membrane. In some cases, the inflammation is confined to the uterus, and it is generally most severe in this organ, or in the parts immediately contiguous. Even when it has extended to the other viscera, and affected them most severely, the peritoneum of the uterus invariably exhibits signs of recent inflammation. The lymph is for the most part thrown out in thicker masses around the uterus, than in any other situation, and this viscus has seemed to suffer in the greatest degree from the violence of the inflammation.

Sometimes considerable depositions of pus are formed in the cellular membrane, beneath the peritoneal coat of the uterus, which are either prominent and circumscribed, or the cellular membrane becomes infiltrated with pus. This latter appearance I have most frequently met with, at the part where the peritoneum is reflected from the uterus and vagina to the rectum.

Inflammation of the peritoneal coat of the uterus is characterized by great tenderness of the surface of the organ, increased on pressure, and by pyrexia more or less severe. In every instance on a careful examination of the uterine region, there has been more or less pain in it increased by pressure, with constitutional disturbance, though it must be admitted that the pain and febrile symptoms have varied greatly in intensity.

When the attack of peritonitis is severe, the patient generally lies upon the back, with the knees drawn up to the trunk of the body. At the onset of the disease, the abdomen is generally soft, and flaccid, and, except in the region of the uterus, not affected by pressure. Dr. Hulme has described the pain as affecting the whole hypogastric region from the commencement of the attack, but this is the case only where the disease has made considerable progress, or has extended from the uterus to the general investing membrane of the abdomen. Though an enlarged and painful state of the uterus be never altogether wanting, yet the pain often undergoes exacerbations, similar to after-pains, and is often mistaken for them by careless observers, and the disease is thus overlooked, till a great part of the peritoneal sac is inflamed, and the case in consequence is rendered hopeless.

The whole abdomen at length becomes distended, tympanitic, and occasionally exquisitely painful on pressure. Vomiting of dark green coloured fluid substances follow. The pulse grows rapid and feeble. The tongue dry and brown, the lips and teeth covered with dark sordes, diarrhœa frequently supervenes, and death follows at no very remote period.

The invasion of pain in the uterus, is sometimes sudden, at other times the ordinary increased sensibility of the uterus, subsequent to the efforts of natural labour, or after-pains, passes slowly and insensibly into the acute pain increased by pressure, which is the great characteristic symptom of uterine inflammation. Most frequently the accession of the disease is marked by rigors, partial or general, sometimes so slight as scarcely to be perceived by the patient, at other times so violent as to produce strong succussions of the whole body. The cold shivering after a longer or shorter duration passes away, and is succeeded by great heat of the surface, acceleration of the pulse and of the respiration, thirst, sometimes nausea, and vomiting, and intense pain across the forehead. The rigors precede, accompany or follow the increased sensibility of the uterus. In some of the most severe cases, there has been no distinct rigor, but a quick pulse, hot skin, and hurried respiration, have rapidly succeeded to the uterine pain. In some of the most unfavourable cases, the extremities have been cold, and the countenance anxious and pallid, after the disease has been completely formed.

There is no uniformity in the state of the tongue in puerperal peritonitis. It is sometimes covered with a thin, moist, white, or cream-like film, at other times it is red in the centre, with a thick, yellow, or white fur on the edges.

The lochia are often completely suppressed, in other cases only diminished in quantity. The mammæ usually become flaccid, yet in some fatal cases, the milk has been secreted till a short period before death." 385.

Puerperal peritonitis may be confounded with those irregular contractions of the uterus which constitute after-pains and hysteralgia; and it must be admitted that, in some cases, it is difficult to draw a line of distinction between them. Where the pulse is accelerated, the remissions of pain incomplete, the lochia scanty or suppressed, in a large proportion of cases, we shall be safe in considering the peritoneal coat of the uterus, or even more deep-seated tissues, as involved in a state of inflammation or congestion, and requiring antiphlogistic treatment. Dr. Lee thinks there are few puerperal women, unless they are of a very feeble and irritable constitution, who can be hurt by cautious depletion under the above circumstances.

"Intestinal irritation, depending on a disordered state of the bowels is also liable to be mistaken for peritonitis, and treated by blood-letting, to the injury of the patient. In this affection the abdominal pain is diffused, it is rather a griping than acute pain; it does not commence in the region of the uterus, nor is it aggravated by pressure. The abdomen is generally soft, puffy and distended. The tongue is loaded. There is thirst and head-ache, the lochia and milk are not suppressed, the febrile attack is usually preceded by evident signs of great intestinal derangement, flatulence, nausea, vomiting, constipation or diarrhœa. The constitutional disturbance attending intestinal irritation comes on about the end of the first week, whereas peritonitis manifests itself most frequently before the fourth day subsequent to delivery. The re-action which succeeds to uterine hæmorrhage cannot easily be confounded with puerperal peritonitis. The morbid sensibility of the uterus which characterises inflammation, and the other symptoms already described, are here entirely wanting." 387.

### II. Inflammation of the Uterine Appendages.

In only one case has our author found the uterine appendages free from disease, where the peritoneal covering of the uterus had been inflamed. On the other hand, the appendages of the uterus have been found extensively disorganized, where the peritoneal coverings have been found very slightly affected.

"The surface of the broad ligaments, ovaria, and Fallopian tubes, have been red and vascular, and partially or completely imbedded in lymph or pus. The loose extremities of the Fallopian tubes have been of a deep red colour and softened, and deposits of pus, in a diffused or circumscribed form, have taken place in their cavities or in their sub-peritoneal tissues. Between the folds of the broad ligaments, effusions of serous or purulent fluids have also been found. Numerous important changes have likewise been observed in the structure of the ovaria. Their peritoneal surface has often been found red, vascular, and imbedded in lymph, without any visible alteration of their parenchymatous structure, or their whole volume has been greatly enlarged, swollen, red and pulpy; blood has been effused into the vesicles of De Graaf, or around them, and circumscribed deposits of pus have been found dispersed throughout the substance of the enlarged ovaria. In several cases, the entire structure of the ovaria has been reduced to a broken-down vascular pulp, no traces of their natural organization being left. These are accurately represented in the drawings now exhibited.

The ovarium appeared, in one instance which I observed, to be converted into a large purulent cyst, which had contracted adhesions with the abdominal parietes, and discharged its contents exteriorly through an ulcerated opening. In another case which proved fatal, the inflamed uterine appendages, agglutinated together by lymph, had contracted adhesions with the peritoneum at the brim of the pelvis, the inflammation had extended to the cellular membrane, exterior to the peritoneum, and had given rise to an extensive purulent deposit in the course of the psoas and iliacus internus muscles, as in lumbar abscess.

In two other individuals, who ultimately recovered, the purulent matter formed in the situation of the psoas and iliacus internus muscles, from inflammation of the uterine appendages, made its way through an opening at the upper part of the thigh. Contraction of the thigh on the trunk took place in both these cases, and continued for several months, but disappeared when complete recovery took place. The uterus remains immoveably fixed to the right side of the pelvis, in a woman who, six months ago, had a severe attack of inflammation of the peritoneum, and uterine appendages of the same side, a few days after delivery." 389.

It is often difficult to form a diagnosis between inflammation of the uterine appendages and peritonitis, because, in fact, they are generally combined together. The pain, it may be said, is less acute than in peritonitis, and it is chiefly seated in one or other of the iliac fossæ, extending thence to the loins, anus, and thighs. On pressure, the morbid sensibility will be found chiefly to exist in the lateral parts of the hypogastrium. The constitutional symptoms, at the onset of the attack, do not differ materially from those which mark the accession of peritonitis, being often accompanied with strong febrile re-action, which passes speedily away, and is succeeded by prostration of strength, and the other appearances which characterize inflammation of the muscular and venous tissues of the uterus. The following cases have been selected to illustrate the morbid changes above described, as appertaining to inflammation of the peritoneal coat of the uterus and its appendages.

" *Case* 1. Mrs. Groom, æt. 28, No. 13, Little Coram Street, was delivered of her first child on the 6th March, 1827. On the 8th, great tenderness of the uterine region took place, with suppression of the lochia, and febrile symptoms, which, being supposed by her medical attendant to depend on spasmodic contractions of the uterus, were treated with anodynes and warm fomentations to the hypogastrium.

On the 10th, (the 4th day after her confinement, and the first on which I saw her) the abdomen was tympanitic and exquisitely painful on pressure. The pulse 140 and feeble, the extremities cold, countenance haggard. There was incessant vomiting of a dark green fluid, with diarrhœa, and she died in the afternoon.

*Dissection.*—The stomach and small intestines were inflated with gas. The peritoneum, covering the fundus and posterior part of the uterus, was of a bright red colour, and the cellular membrane underneath it, in this latter situation, was infiltrated with pus. The peritoneal coat of the small intestines was highly vascular in different parts, and the surface of the liver was partially covered with lymph. The uterine appendages on both sides were covered with pus and lymph, and the lumbar regions contained about a pint of a wheyish-coloured turbid fluid. The consistence of the spleen was remarkably soft.

*Case* 2. Elizabeth Marshall, æt. 23, No. 3, Crown Place, Soho. Was attacked on the 4th of March, 1827, (the 3d day after her delivery) with rigors, head-ach, vertigo, and sense of exquisite tenderness in the hypogastrium and right groin. The milk and lochia soon disappeared, blood-letting was employed on the 8th, and leeches were applied to the region of the uterus, but the tenderness gradually extended over the whole abdomen, which became as large as before delivery, and tympanitic. The pulse was rapid and intermitting. The tongue covered with a brown fur, singultus and vomiting of dark-coloured matter succeeded, and she died on the 12th day after the attack.

*Dissection.*—The uterus with its appendages, and the small intestines, were all imbedded in thick masses of lymph, and closely adhered to one another. The omentum, colon, and peritoneum lining the abdominal muscles were vascular, of a deep red colour, and partially coated with false membranes. About ℥x. of sero-purulent fluid were contained in the cavity of the abdomen. The deeper-seated tissues of the uterus were healthy.

*Case* 3. Mrs. Laurens, æt. 42, at No. 5, Cumberland Street, Middlesex Hospital.

After a severe and protracted labour, was delivered of a still-born hydrocephalic child, on the 12th of February, 1828. On the 14th, there was a severe rigor, the lochial discharge was suppressed, and the uterus was felt above the brim of the pelvis, large, hard, and exquisitely painful on pressure. The pulse 120, with great prostration of strength.

On the 15th, the pulse was more rapid and feeble, the abdomen tumid, and every where highly sensible. Vomiting of green-coloured matter took place, and she died about 60 hours from the period of delivery.

*Dissection.*—The uterus uncontracted occupied the whole brim of the pelvis; its peritoneal coat, and that of the small intestines and liver was partially covered with thin false membranes, and two pounds of a brownish-coloured fluid, with flakes of albumen and pus, were contained in the peritoneal sac. A fibro-cartilaginous tumour, of considerable size, was found imbedded in the muscular coat of the uterus. The uterine appendages on the right side were red and vascular, and the ovarium was unusually soft, and about three times the natural size.

*Case* 4. Mrs. Tiffin, æt. 32, No. 18, Mercer Street, Long Acre.

Delivered on the 7th July, 1829. Labour natural. On the 9th, the uterus was felt above the brim of the pelvis, large and hard, and it was very painful on the slightest pressure; lochia and milk suppressed; pulse 110, and feeble; tongue white; bowels open. Slight relief followed the abstraction of fifteen ounces of blood from the arm, and the application of leeches to the hypogastrium.

10th July. The whole hypogastrium is now exquisitely painful, and the abdomen is swollen. Pulse more frequent. There has been much nausea and vomiting during the night. Bowels open. F. V.S. ad ℥xxiv. Eighteen leeches to the region of the uterus.

11th. Vomiting continues, abdomen less swollen, and pressure over the region of the uterus produces little uneasiness. Pulse rapid and feeble, respiration hurried, countenance sunk, occasional delirium. The whole surface of the body is now of a deep yellow colour.

She became gradually more feeble, and died in the evening.

*Dissection.*—Present, Drs. Sims, Clark, and Williams. The abdomen was distended by a great accumulation of air within the bowels, the peritoneal coat of the small intestines was red and vascular: the peritoneum of the fundus and anterior portion of the body of the uterus was coated with albumen, and the sub-peritoneal tissue, in this situation, contained a sero-purulent and gelatinous fluid. From the incisions made into the lower part of the body of the uterus, there escaped pure pus, but whether this flowed from the vessels or muscular tissues it was not easy to ascertain. Between the folds of the broad ligaments, there was a deposition of a gelatinous and purulent fluid, and both Fallopian tubes were of a deep red colour, softened, and their coats filled with pus. The right ovarium was of the size of a common hen's egg, of a pulpy gelatinous consistence, and its healthy organization entirely destroyed. The whole presented the appearance of a soft, fibrous, vascular pulp; the left ovarium was similarly affected.

*Case* 5. Mary Ann Hale, æt. 26, was delivered in the British Lying-in Hospital, on the 24th July, 1829. On the 26th she had a severe rigor, which was speedily followed by pain in the region of the uterus and febrile symptoms: 18 ounces of blood were drawn from the arm, which produced but little relief: leeches and other antiphlogistic remedies were employed: the whole abdomen, however, soon became exquisitely tender, without swelling or tension, and death took place on the 29th, the 5th day after delivery. Cough, dyspnœa, and pain in the right side of the chest, were experienced during the two last days of her life.

*Dissection.*—The peritoneal coat of the uterus, and the uterine appendages, were coated with false membrane; that covering the small intestines exhibited the usual effects of intense inflammation. Several folds of the ilium were glued together by lymph. The surface of the liver was also coated with albumen, and about two pounds of a whey-coloured fluid were contained in the abdominal cavity. The muscular coat and vessels of the uterus were in a healthy condition. In the left side of the thorax there were traces of recent inflammation in the pleura and substance of the lungs." 396.

Four other cases are related by Dr. Lee, but we conceive the five foregoing instances are quite sufficient for our purpose in this place. We shall, therefore, proceed to the third division of the subject.

### III. INFLAMMATION AND SOFTENING OF THE MUSCULAR TISSUE OF THE UTERUS.

The dark-coloured layer, says our author, which usually coats the inner surface of the uterus, after delivery, has been supposed to be the result of gangrenous inflammation, and has been described as such, by some pathologists. This ought not, however, to be confounded with the changes produced by inflammation of the inner membrane of the uterus, when it becomes softened or wholly disorganized like the mucous membrane of the stomach and intestines in certain inflammatory affections.

"Inflammation and softening of the uterus have in some cases affected the muscular tissue of the fundus, body, and cervix of the uterus; in others these changes have been limited to the part where the placenta has adhered, which has become unusually thin and reduced to a pulpy state.

Small abscesses have been formed in a few instances in the proper tissue of the uterus, without any perceptible change in the surrounding substance of the organ, while in other cases all appearance of muscular fibre has been lost.

In the works of the different authors on puerperal fever published in this country, the rapid and destructive variety of uterine inflammation now described has scarcely been noticed, though it has been pointed out by several German and French pathologists. Astruc, Vigarous, and Primrose state, that the uterus is liable to be attacked with gangrene and sphacelus; and other authors have recorded cases where gangrene of the uterus followed acute inflammation of the organ. Professor Boër, of Vienna, has described this affection under the term Putrescence of the Uterus, and has observed its frequent occurrence in particular epidemics.* Luroth,† and Danyau,‡ have more recently published extended accounts of this malignant affection, which occurs soon after delivery, and often runs its course with great rapidity. Among the 222 fatal cases of puerperal fever observed by M. Tonellè in the Maternité of Paris in the year 1829, there were forty-nine in which the muscular tissue of the uterus was found softened. M. Tonellè states, that softening of the uterus, after shewing itself frequently in the first half of the year 1829, and particularly about January, disappeared entirely in the months of July and August, which were characterized in a remarkable manner by the frequency of uterine phlebitis. Afterwards it began to rage anew with great violence in September and October, and disappeared again in the two last months, during which time the mortality was inconsiderable.

Considerable obscurity, as has been well observed, exists, respecting the ordinary effects of inflammation of the muscular fibres of the body, but one point may be considered as known and established, viz. that a result of inflammation in this tissue of the body is softening and gangrene.§ That the destruction of the healthy organization of the proper tissue of the uterus, in puerperal women, is the consequence of an inflammatory process, may be inferred from the symptoms which accompany the disease, and from its occurring in combination with the other varieties of uterine inflammation.

Inflammation of the muscular coat of the uterus, most frequently commences with pain of the hypogastrium, irregularity of the lochial discharge, and rigors, succeeded by the other symptoms of pyrexia. The countenance becomes pallid, and is usually expressive of great anxiety and distress. There is often severe head-ache, with delirium and other affections of the brain and nervous system, and so violent have these been in some cases, that the local affection of the uterus has completely escaped detection during life. The skin is hot and dry, and sometimes of a peculiar sallow tinge, the pulse is rapid and feeble. The respiration hurried, with remarkable prostration of strength. The tongue soon becomes foul. The lips covered with sordes: occasional vomiting is experienced. The progress of the disease in some cases is rapid, in others it runs its course more slowly, being protracted to the eighth or tenth day." 405.

The diagnosis between this variety of uterine inflammation and peritonitis or phlebitis, with which it is often combined, is acknowledged to be very difficult, or even impossible. In all the cases of this affection which our author has seen, the resources of Nature and Art have proved of no avail. The active inflammatory symptoms which commonly manifest themselves at the commencement of the attack, pass speedily away, whatever plan of treatment be adopted, and are rapidly succeeded by symptoms of exhaustion. When the disease is not complicated with peritonitis, the symptoms are not so urgent as to indicate venesection—and in one case where it was adopted freely, the abstraction of blood was followed by speedy death. In other cases, and where opposite plans were followed, the fatal result was not less certain, though somewhat more slow. The three following cases are adduced as illustrations of the disease in question.

---

* "Naturalis Medicin. Obstetric. Libri VII. Viennæ, 1812."

† "Mémoire sur le Ramollissement. Par G. S. Luroth. Repertoire Generale d'Anatomie Pathologique. 1828."

‡ "Essai sur la Metrite Gangreneuse. Par A. C. Danyau. Paris, 1829."

§ "Pathological and Practical Researches on Diseases of the Stomach. By J. Abercrombie, M. D. Edin. 1828."

*Case* 1. "Mrs. D——, Orange Street, Leicester Square, after a severe and protracted labour was delivered of a still-born child, on the 25th of March, 1829. On the 27th, there was exquisite tenderness of the hypogastrium increased by pressure, with fulness and tension of the whole abdomen. The pulse was rapid and feeble. The lochia and milk suppressed. The tongue was dry and furred. Thirst urgent, with constant nausea. Leeches and warm cataplasms were applied to the region of the uterus, and calomel and opium administered every second hour. The pain gradually extended to the whole abdomen, which was enormously distended. The pulse became still more rapid and feeble. The tongue brown; teeth covered with dark sordes. Incessant vomiting of dark coloured matters, with low muttering delirium, followed, and she sunk on the 4th of April.

*Dissection.* The peritoneal surface of the great intestines was remarkably vascular, but no false membrane was observed on any of the abdominal viscera. Several pints of a brown serous fluid were contained in the peritoneal sac; the uterus was large and uncontracted, and its peritoneal coat at the inferior and posterior part was deeply red; its muscular tissue to a considerable extent in this situation, was of a dark ash-grey colour, and so soft as to be lacerated by slight pressure of the fingers. The os uteri at the posterior part was softened and wholly disorganized.

*Case* 2. On the 7th of September, 1829, I was present at the examination of the body of a lady who had died on the 9th day after delivery, with the ordinary symptoms of low child-bed fever.* Little complaint had been made of pain in the region of the uterus. The pulse was rapid and feeble, the respiration hurried, the tongue loaded, with diarrhœa. Before death the whole surface of the body had assumed a deep yellow colour.

*Dissection.*—The uterus occupied the brim of the pelvis. The whole peritoneal sac had a healthy appearance, except a small portion covering the posterior part of the body of the uterus, which was red and vascular, but not covered with false membrane. On cutting into the cavity of the uterus, there escaped a dark coloured offensive fluid. The muscular coat under the inflamed peritoneum, where the placenta had adhered, was converted into a soft flocculent substance, readily broken down with the fingers, and this morbid alteration extended near to the peritoneum. Around this disorganized portion of the muscular and internal coats of the uterus, similar changes, though slighter in degree, were observed in these tissues to a considerable distance, and they had a dark livid colour.

The uterine appendages on the right side were also disorganized by inflammation.

*Case* 3. Mrs. Chapman, æt. 36, No. 9, Belton Street, Long Acre. Delivered on the 19th of August, 1830, labour easy. On the 24th, after drinking freely of porter, was suddenly attacked with a violent rigor, of long continuance, which was succeeded by acute uterine pain, headache, and great frequency of pulse. No remedies of any kind were employed until the 27th, when I was first called to see her. Ske had been delirious in the night. The pulse 130, soft and compressible; hurried breathing, great prostration of strength. Tongue brown and furred, diarrhœa, surface of the body of a deep sallow colour. The hypogastrium was painful on pressure, the abdomen generally neither swollen nor tender.

The symptoms became aggravated in the night, and she died on the morning of the 28th.

---

* "My friend and colleague, Dr. Henry Davies, was consulted in this case, and it was to his kindness that I enjoyed the opportunity of witnessing this dissection, and of examining the bodies of several other women who had died in the hospital."

*Dissection.*—Dr. Sims and Mr. Rice were present. No trace of disease could be detected in the peritoneal coat of the uterus, intestines, or other abdominal viscera, and no effusion of fluid had taken place into the peritoneal cavity.

Both ovaria were enlarged and disorganized, being so softened in consistence as to resemble a rotten pear. Both Fallopian tubes were of a deep red colour, and their cavities were filled with a thin purulent fluid. These morbid appearances were most remarkable in the right uterine appendages. The muscular coat of the greater portion of the body and fundus of the uterus, at the posterior part, was of a peculiar yellow colour, and so soft, that the point of the fore-finger passed through it and the peritoneum covering it, though the parts were dissected out in the gentlest manner. On a careful examination of the uterus, it was found that the whole of the uterine parietes at the posterior part had undergone this morbid change of structure." 411.

IV. The next subject which our author investigates is Inflammation of the Veins and Absorbents of the Uterus. A case was observed by Mr. Cæsar Hawkins, at St. George's Hospital, where the absorbent vessels of the uterus, and the receptaculum chyli were filled with pus, in a case of fatal uterine inflammation after delivery. Since that period, our author has observed the absorbents in the vicinity of the uterus distended with pus in four cases; and in three of these there existed inflammation and suppuration of the veins. The late valuable researches of Tonellé and Duplay have proved that inflammation of the uterine absorbents, of the receptaculum chyli and thoracic ducts, occurs not unfrequently in puerperal women, and gives rise to the same constitutional disturbance as uterine phlebitis. The presence of purulent fluid in the veins of the uterus after parturition was pointed out many years ago by Meckel, Wilson, Clarke, and others; but none of these authors appear to have been aware of the important fact, recently demonstrated, that a large proportion of what are termed low child-bed fevers, arise from inflammation and suppuration of the uterine veins. Exclusive of the cases published in the 15th volume of Med. and Chir. Transactions, ten fatal examples of this insidious and most dangerous affection have fallen under his notice since November 1829; and, from an examination of all these cases, it appears to him, that "the symptoms of uterine phlebitis correspond, in a striking manner, with the symptoms assigned by the earlier writers to the putrid puerperal fever, or malignant forms of typhus after delivery."

M. Tonellé, in his memoir, has stated that, in 1829, during the prevalence of the fatal epidemic in the Maternité, inflammation of the veins and lymphatics of the uterus occurred in 132 out of 222 cases which were examined after death; and that, in 197 cases of the whole, some important alteration of structure was discovered in the uterine organs. M. Duplay has confirmed these observations to the fullest extent; for he met with eighteen cases of inflamed lymphatics, with or without phlebitis. In all of these the constitutional phenomena were those which characterize inflammation of veins in other parts of the body, and in the other sex.

"In women who have enjoyed good health during pregnancy, and in whom the process of parturition has been easily accomplished, uterine phlebitis occasionally commences within twenty-four hours after delivery, with pain more or less acute in the region of the uterus, accompanied or followed by a severe rigor, or a succession of rigors, suppression of the lochial discharge, acceleration of the pulse, cephalalgia or slight incoherence of ideas, with an insuperable sensation of general uneasiness, and sometimes by nausea and vomiting. These symptoms after a short duration are succeeded by increased heat of the body, tremors of the face and limbs, rapid feeble pulse, anxious and hurried respiration, great thirst, with brown dry tongue, and frequent vomiting of green-coloured matters. The sensorial functions usually become much affected, and there is a state of drowsy stupor or violent delirium and agitation, which terminate in exhaustion. The whole surface of the body not unfrequently assumes a peculiar sallow or deep yellow colour, the abdomen becomes swollen and tympanitic, and some of the remote organs of the body, the brain, heart, lungs, liver, and spleen, or the articulations and cellular membrane of the extremities suffer disorganization, from a rapid and destructive congestion, inflammation, or gangrene.

At other times, inflammation of the uterine veins commences at a later period after delivery than above-mentioned, and in a much more obscure and insidious form, without either pain or sense of uneasiness in the region of the uterus, or any other local symptom by which the affection can be recognized. The uterus may return to its usual reduced volume after delivery, the lochial discharge may continue to flow, and the inflammation and suppuration of the veins, which have caused the whole of the violent constitutional disturbance and destructive lesions in distant parts of the body, may be wholly overlooked during life. In several cases which I shall now relate, this occurred, and wine, opium, brandy, and sulphate of quinine, with other stimulants, were liberally administered by the medical attendants, to obviate the debility supposed to be caused by a specific fever, without any local affection of the uterine organs." 416.

Phlebitis rarely takes place in any part of the body where it cannot be referred to a wound, or to some specific cause externally applied. In uterine phlebitis the inflammation cannot, it is true, be traced invariably to the orifices of the veins where the placenta adhered to the inner surface of the uterus; "yet it scarcely admits of a doubt, says Dr. Lee, that the frequent occurrence of the disease is the effect of the communication indirectly established between the venous system and the atmospheric air after the separation of the placenta." In consequence of this separation, Dr. Lee conceives that the uterine veins are placed in a condition analogous to that of the great veins of the extremities after amputation and extensive wounds, which condition, experience has proved to be favourable to the production of inflammation. Inflammation once excited in these vessels may extend along the continuous membrane of the uterine veins to the spermatic or hypogastric veins, and thence to the vena cava and its principal branches, returning the blood from the lower extremities.

Though uterine phlebitis is a most dangerous affection, it does not always prove fatal. It often occurs, Dr. Lee thinks, in puerperal women, without being suspected. We are now prepared for the detail of some cases of—

### Uterine Phlebitis.

*Case* 1. "Mrs. Hickson, a middle aged woman, delivered in the British Lying-in Hospital, on the 14th of November, 1829. On the 3d of December, the day before her death, I first saw her. The hypogastrium was swollen and tense, and on the right side exquisitely painful on pressure. The pulse was 130 and feeble; respiration hurried. The countenance sunk; great prostration of strength. The tongue covered with a dark brown fur; nausea, and urgent thirst. The conjunctiva of both eyes, and the whole surface of the body of a deep yellow tinge. The milk, which was sparingly secreted, was observed to be of the same colour. I was informed that this patient had a very good labour, but that retention of urine took place a few days after she had complained of some pain in the right side, which was relieved by leeches. She afterwards went on tolerably well, and was up and about till the middle of the third week. She took porter and animal food eagerly till within two days of her death.

The body was removed from the Hospital to Little Brook Street, Hanover Square, where it was examined on the 8th of December.

The peritoneal surface of the abdominal viscera appeared at first sight in a healthy state, and the uterus had undergone the usual reduction of volume, at the same period after delivery. The uterine appendages on the right side were found adhering to the caput coli and to the peritoneum near the brim of the pelvis, by a firm false membrane. The veins proceeding from the right side of the fundus uteri to the spermatic were filled with pus, and the coats of the right spermatic veins, to an extent of three inches from the uterus were greatly thickened, and the cavity obstructed with lymph and pus. The veins in the left superior angle of the uterus also contained pus, and two small purulent deposits were found immediately under the peritoneum in the same situation.

Upwards of a pint of pure pus was contained in the cellular membrane at the brim of the pelvis on the right side, and had passed down into the cavity exterior to the peritoneum, as low as the neck of the bladder. The mucous membrane of the bladder near its cervix was intensely red, and partially coated with a thin false membrane of an ash-grey colour.

*Case* 2. Mrs. Messlin, æt. 22, a patient of the British Lying-in Hospital, delivered on the 13th of January 1830, after a natural labour. During the whole of the following day she complained of an unusual sense of chilliness, with vertigo and slight head-ache.

15th Jan. She now complains of acute pain in the left side of the chest, with confined respiration and cough. There is also great tenderness in the region of the uterus, the body of the uterus is felt above the brim of the pelvis, large, and hard, and pressure over it produces exquisite suffering. Pulse above 100, full, and soft. Countenance flushed; skin hot. Lochia and milk suppressed. V. S. ad ℥xvj. Hirud. xxiv. Calomel and opium every second hour.

16th. The uterine pain was immediately relieved by the bleeding, but it returned again in the night, when fourteen ounces more were drawn from the arm.

In the afternoon the abdomen was considerably distended, but soft. The uterus still large, hard, and painful on pressure. Pulse rapid and feeble; great prostration of strength. Has been drowsy and oppressed since the morning, and makes no complaint but of distressing sickness at stomach.

During the 17th, the abdomen became more distended; the pulse more rapid and feeble, and she sunk on the morning of the 18th, the fifth day after delivery.

*Dissection.*—The lungs on the left side gorged with blood, pleura healthy. The caput coli and transverse arch of the colon were preternaturally vascular, and here and there covered with patches of lymph. The uncontracted uterus filled the brim of the pelvis. The peritoneum of the anterior part of the fundus and body of the uterus was of a dusky red colour, and the veins at both superior angles of the uterus were gorged with pus. The spermatic and hypogastric veins on both sides were healthy. The muscular tissue at the anterior and superior part of the uterus, where the placenta had adhered, was reduced to a soft, red coloured, flocculent pulp.

Both ovaria were much enlarged, vascular, soft, and their parenchymatous structure infiltrated with pus and lymph. Both Fallopian tubes were of a red colour, and contained pus in their cavities.

On the 16th of January, three days after the occurrence of the last case, another patient in the hospital was attacked the day after delivery with rigors, head-ache, and great tenderness of the uterus, with diminished lochial discharge. The pulse was 110, and weak; skin hot; the countenance pale and depressed. The abstraction of ℥xx. of blood from the arm, and the application of twenty-four leeches to the hypogastrium were followed by immediate relief of all the symptoms.

Another case occurred on the same day, which yielded to similar treatment.

*Case* 3. On the 19th of January, 1830, with Mr. North of Upper Berkeley Street, I examined the body of a woman in Portman Mews, who had died twelve or fourteen days after delivery. It was stated by her medical attendant that the labour had been natural, and that she continued well till the fifth or sixth day after delivery, when tenderness of the abdomen came on, with fever, which soon assumed a low typhoid type. The pulse was rapid and feeble, and the tongue brown and parched. Sulphate of quinine and stimulants were liberally administered, but the symptoms assuming a more unfavourable character, Mr. North was called to see her. A puffy swelling of considerable magnitude had appeared over the left wrist, and another in the right thigh, about the middle.

*Dissection.*—A copious sero-purulent effusion into the abdominal cavity. The uterus larger than usual at the same period after delivery. The peritoneum, covering its anterior part, highly vascular, and covered with a thick albuminous layer. The veins proceeding from the left superior angle of the uterus, left ovarium, and Fallopian tube were fully distended with a purulent sanious fluid. The coats of the left spermatic vein, throughout its whole course, were greatly thickened and contracted; the lower half of the inner surface of the vein was lined with false membranes, and the cavity partially filled with pus. The superior half was blocked up with firm coagula of blood. The muscular tissue of the fundus uteri to a considerable extent on the left side was of a dull yellow colour, but the part preserved its natural consistence. The veins on the right superior portion of the uterus were filled with pus. The right spermatic and both hypogastric veins were healthy." 423.

We do not deem it necessary to quote any more of the cases of uterine phlebitis, since they would only be repetitions of what have already been adduced; but the following case of inflammation of the ABSORBENT VESSELS should here find a place, in order that the chain of our analysis may not be broken.

*Case.* "Mrs. Wall, æt. 32, No. 89, Berwick Street. Delivered of her second child on the 1st of Nov., 1830. Labour protracted from deformity of the brim of the pelvis. On the morning of the 2d of November, the day after delivery, she was attacked with acute pain of the uterus, with complete suppression of the lochia, and febrile symptoms. The uterus could be felt preternaturally large and hard in the hypogastrium, and very tender on pressure. The other parts of the abdomen were soft and flaccid, and not affected by pressure. The pulse was 100, soft and compressible. A pint of blood taken from the arm was followed by syncope and great relief of uterine pain. Eight leeches were applied to the hypogastrium, and calomel and antimonial powder administered every fourth hour. Warm cataplasms were applied over the leech-bites.

3d November. Pain of uterus now produces little uneasiness, except when pressure is made over the hypogastrium. The uterus can still be felt unusually large and hard above the brim of the pelvis. Pulse extremely rapid and feeble. Countenance pale and dejected. She is now affected with somnolence to so great a degree that she can scarcely be roused.

She became gradually more feeble and sunk in the night.

*Dissection.*—Two pints of a dark brown serous fluid in the sac of the peritoneum. The right ovarium enlarged to the size of a hen's egg, the surface of a bright red colour, and imbedded in lymph, its structure disorganized, the whole presenting the appearance of a soft cyst, distended with a purulent and gelatinous fluid. The left ovarium had lost all traces of its natural form and texture, being reduced to a broken down flocculent pulp. The absorbents of the uterus, on the left side and in the left broad ligament were filled with pus. The veins and muscular structure were healthy.

The appearances of the ovaria in this case have been faithfully represented in the drawings presented to the Society." 429.

From the time the British Lying-in Hospital was opened, in the Summer of 1830, no case of uterine inflammation occurred till the month of December, when three fatal cases were observed. We shall give a very condensed outline of these.

*1st. Case.* Was a female aged 30, who was delivered on the 19th Dec. without any difficulty. On the 21st she had a severe rigor, followed by tenderness in the uterine region, head-ache, and suppression of the lochia, fever, &c. Bled to 20 ounces, and had three grains of calomel, with a quarter of a grain of opium every four hours. Thirty-six leeches to the hypogastrium. 22d. Very little alteration. 23d. Abdomen enormously distended, tympanitic, and painful. Pulse rapid—thirst urgent—somnolency—delirium. She died in the night. Permission could not be obtained to open the body.

*Case* 2. Mrs. Jones, æt. 24, was attacked 24 hours after delivery, with sickness, head-ache, and rigors. The lochia were suppressed—pain in the hypogastria—collapse of the features—pulse 120 and feeble. These symptoms were exasperated, and she died on the 24th.

*Dissection.*—"The placenta had been attached to the left side of the fundus uteri, and the veins at this part of the uterus were lined with dark coloured false membranes, and gorged with pus. The lymphatics of the left broad ligament were distended with purulent fluid. Both ovaria were enlarged, and reduced to a soft flocculent pulp.

The Fallopian tubes were both red and vascular, and their cavities full of pus.

The peritoneal coat of the uterus at the posterior part was inflamed, and about four ounces of yellow serum were effused into the pelvis. A few inflamed patches were observed on the peritoneal surface of the small intestines." 431.

*Case* 3. C. Boyd, æt. 31, was admitted on the 25th Dec. for labour pains; but as they were deemed spurious, she returned home till the 28th, when they became violent, and she was delivered at her own residence. The labour was natural. On the 31st Dec. she was attacked with pains in the uterus, rigors, and occasional delirium, having a rapid feeble pulse, and pallid countenance. The abdomen was tumid and soft. The hypochondrium and iliac fossæ were tender. Jan. 1st. Complete remission of pain, except on firm pressure over the region of the uterus. Constant dozing—pulse 140—tongue brown and dry in the centre. 2nd. Much the same. 3d. Seized with excruciating pain of the abdomen, with distressing flatulence. Abdomen distended—pulse rapid, feeble, and irregular. She died on the 4th. The abstraction of eight ounces of blood from the arm, at the onset of the disease, produced complete syncope. Mercurial frictions, with calomel and opium internally, were largely employed.

*Dissection.* "Abdomen distended with gas. Six ounces or more of red serous fluid in its cavity. Peritoneal sac not inflamed, except that portion covering the posterior surface of the uterus and its appendages. The cellular tissue connecting the peritoneal with the muscular coat, at the back of the cervix uteri infiltrated with pus, as well as that between the folds of the broad ligaments, on both sides. Both spermatic veins contained pure pus in considerable quantities, as did also the venous branches, at the angles and inferior portions of the uterus. The Fallopian tubes enlarged and vascular. The muscular structure of the uterus healthy. No appearance of pus was observed in the orifices of the veins, at the part to which the placenta had been attached." 433.

### ON THE CAUSES OF UTERINE INFLAMMATION.

These are generally very obscure. In some cases the disease is distinctly referrible to injury inflicted on the uterus by protracted or instrumental labour. But most frequently it arises where none of these causes have been applied, and where we are compelled to refer it to some peculiar constitution of the atmosphere, or to contagious miasmata.

"It is a point of the utmost practical importance to determine, how far contagion is to be considered as a cause of the disease; the writers on puerperal fever are, however, completly at variance on this subject. Dr. Hulme maintains that it is not more contagious than pleuritis, nephritis, or any other inflammatory disease, and M. Tonellé, who has recorded the history of the most fatal epidemic which has ever occurred in Paris, asserts that contagion was clearly out of the question there, for, in the Maternité, the women who were newly delivered had each a separate apartment, and yet were attacked with the disease, while, in the sick ward of the hospital, no instance of the propagation of puerperal fever ever occurred." 436.

The evidence of M. Dugés against contagion is still more strong. The sentiments of Baudelocque are in unison with those we have mentioned. In the great hospitals, however, of Dublin, Edinburgh, Vienna, and London, it has appeared to be contagious. Dr. Gordon, Dr. Armstrong, and Dr. J. Clarke are contagionists. The latter, indeed, says "it is hardly possible to prove that it is not infectious; but it has arisen also, as far as we can judge, as an original disease in private practice, where there had been no communication with infected persons." This we imagine will apply to more diseases than puerperal fever. Dr. Lee himself is doubtful on the question of contagion. He tells us that, "in many cases, it has occurred in the most destructive form where the idea of contagion could not be entertained." On the other hand, he observes—

"In the last two weeks of September, 1827, five fatal cases of uterine inflammation came under my observation. All the individuals so attacked had been attended in labour by the same midwife, and no example of a febrile or inflammatory complaint of a serious nature occurred, during that period, among the other patients of the Westminster General Dispensary, who had been attended by the other midwives belonging to the institution.

On the 16th of March, 1831, a medical practitioner, who resides in a populous parish in the outskirts of London, examined the body of a woman who had died, a few days after delivery, from inflammation of the peritoneal coat of the uterus. On the morning of the 17th of March, he was called to attend a private patient in labour, who was safely delivered the same day. On the 19th, she was attacked with the worst symptoms of uterine phlebitis; severe rigors, great disturbance of the cerebral functions, rapid feeble pulse, with acute pain of the hypogastrium, and peculiar sallow colour of the whole surface of the body: she died on the fourth day after the attack, the 22d of March, and between this period and the 6th of April Mr. —— attended two other patients, both of whom were attacked with the same disease in a malignant form, and speedily fell victims to it.

On the 30th of March, it happened that the same gentleman was summoned to a patient, a robust young woman, seventeen years of age, affected with pleuritis, for which venesection was resorted to with immediate relief.

On the 5th of April there was no appearance of inflammation around the puncture which had been made in the median basilic vein, but there had been pain in the wound during the two preceding days. The inner surface of the arm, from the elbow nearly to the axilla, was now affected with erysipelatous inflammation. Alarming constitutional symptoms had manifested themselves; the pulse 160, tongue dry. Delirium had been observed in the night.

On the evening of this day, the inflammation had spread into the axilla. The arm was exquisitely painful, but in the vicinity of the wound, which had a healthy appearance, the colour of the skin was natural, and no hardness nor pain was felt in the vein above the puncture.

On the 6th, patches of erysipelatous inflammation had appeared in various parts of the body, the upper and inner surface of the left arm, and in the sole of the left foot, all of which were acutely painful on pressure. The inflammation of the right arm had somewhat subsided. The pulse was 140. The tongue brown, dry, and furred. Restlessness, constant dozing, and incoherence; when roused, she was conscious. The countenance cold, heat of the surface irregular.

The 7th, pulse rapid, countenance anxious, teeth and lips covered with sordes, somnolence and delirium. The left arm above the elbow was acutely painful and very much swollen. The right was but little painful, and the erysipelas had made no further progress. The patches of erysipelas on the forehead and sole of the foot had disappeared, but there was a slight blush of inflammation on the inner side of the calf of the left leg. The symptoms became aggravated, and she died on Saturday, the 9th April.—I examined the body with Mr. Prout on the 11th, and the following morbid appearances were observed.

The wound in the median basilic vein was open, and its cavity was filled with purulent fluid. The coats of this vessel and of the basilic vein, to its termination in the axillary vein, were thickened, so as to resemble the coats of an artery. The inner surface of these veins was redder than natural, and at the upper part had lost its usual smoothness, but there was no lymph deposited upon it. The mouths of the veins entering the basilic were all closed up with firm coagula of blood or lymph. The cellular membrane along the inner surface of the arm was unusually vascular, and infiltrated with serum. This infiltration was to a much greater extent along the situation of the erysipelatous inflammation of the left arm, but the veins of this arm were perfectly healthy. The abdominal viscera were sound." 441.

But, whatever view we take, as to the contagious or non-contagious nature of the disease, Dr. Lee thinks that his doctrine of the proximate cause cannot be affected; since the symptoms, morbid anatomy, and remedies all prove that, whatever be the nature of the remote cause, "it acts by exciting inflammation of the uterine organs." With regard to the nature of the inflammation, it is difficult to determine whether it be of a common or of a specific kind. "It certainly (says Dr. Lee) arises where individuals are not exposed to the ordinary causes of inflammation, and it often reigns as an epidemic, particularly in hospitals; and in this respect it resembles hospital gangrene, erysipelas, and other specific inflammatory diseases, which are generally supposed to depend on a vitiated state of the atmosphere. Like these diseases, too, it ceases without any assignable cause perhaps for several years, and then re-appears in the same establishments, and is attended with the same destructive consequences."

Pouteau believed the uterine inflammation to be of an erysipelatous nature, and the same opinion was entertained by Drs. Home, Young, Abercrombie, and others; but pathologists and physicians are very far from being agreed upon this point; and it is doubtful if the serous membranes be liable to erysipelatous inflammation. Dr. Hodgkin, the able anatomist, avers, that the appearances after death, in puerperal peritonitis, do not differ from those observed in ordinary peritonitis of the male sex.

"In the Autumn of 1829, a short time before the epidemic broke out in the British Lying-in Hospital which led to its being closed for several months, two children died of erysipelas. In one of these which I examined after death, there were inflammation and suppuration of most of the branches of the umbilical vein, and extensive peritonitis. Another fatal case occurred in the course of the epidemic, and on examining the abdomen I found the peritoneum extensively inflamed, with a copious effusion of sero-purulent fluid. A few days before the re-appearance of the disease in the hospital in December last, an infant died of erysipelas of the external organs of generation and abdomen, and the same diseased state of the peritoneum was observed. Another infant was attacked with gangrenous erysipelas of the extremity of the right fore-finger on the 28th of December, whose mother had been cut off on the 24th by uterine phlebitis. Mr. Blagden has related to me a similar case which occurred in his practice last

Summer. A midwife of the hospital had a severe attack of erysipelas of the face, a few days after attending in labour one of the fatal cases I have related of inflammation of the absorbents and uterine appendages. No. XVIII. These are certainly remarkable coincidences, but they are not sufficient I conceive to establish the fact, that it is an erysipelatous inflammation which attacks the uterus subsequent to delivery." 444.

At the close of this paper Dr. Lee has placed an abstract of 112 cases of uterine inflammation, by which it will be seen that, at one period, the inflammation affects chiefly the peritoneal surface of the uterus, whilst at another it affects its deeper-seated tissues, resembling, in this respect, some other inflammatory diseases when they assume an epidemic form.

"It may also be observed from an examination of this abstract, that in the course of a few days, in the same ward of the hospital, and in patients who were placed in contiguous beds during the prevalence of the epidemic, all the varieties of uterine inflammation which I have described, occurred in their most perfect forms. In some the local and constitutional symptoms were immediately subdued by general and topical blood-letting, but in other cases the symptoms were from the commencement such as to contra-indicate the use of this remedy, and it was not had recourse to. Such cases usually terminated fatally in spite of local bleeding, and the exhibition of internal remedies, and on examination after death the veins, muscular structure, or appendages of the uterus, were found to be the textures most frequently inflamed.

This fact, that at different seasons, different textures of the uterine organs are liable to be affected with inflammation, and in varying degrees of intensity, will enable us in some measure to reconcile the discordant opinions contained in the works of authors, both with respect to the symptoms of puerperal fever, and the treatment required in different epidemics." 445.

The pathology of the disease in question is indeed a puzzler; yet the prevailing sentiment is evidently in favour of inflammation in the abdomen or pelvis as the essential feature of the malady.

"Pinel, Bichat, Laroche, and Gardien, found the peritoneum inflamed in so many fatal cases of puerperal fever, that they have considered it to depend essentially on peritonitis. An eminent French author who has subsequently observed the disease, and who entertains the same views of its inflammatory nature, declares that nothing can be more absurd, more chimerical, or contrary to the spirit of analysis and observation, than the idea of a puerperal fever, that is to say, a fever essential or peculiar to a woman recently delivered.

The bodies of fifty-six women were examined who had died in the General Hospital at Vienna in the Autumn of 1819 of puerperal fever, and in all of these, with the exception of two cases, where delivery had taken place some time previous to death, effusions of sero-purulent fluid were found in the abdominal cavity, and traces of inflammation in one or more of the abdominal viscera. The ovaria and Fallopian tubes were always more or less swollen, red, and tender, and the body of the uterus was always, in consequence of inflammation, flabby, tender, and easily broken down with the finger. It is also stated in the report of the epidemic, that the accession of fever is always preceded by marked changes in the whole system and particularly in the uterus, clearly indicating an inflammatory state. The symptoms were such that the inflammation combined with high fever could not be mistaken.*

---

* "Medical Annals of the Austrian States, 1822."

If we consult the works of the most celebrated writers in this country on puerperal fever, it will clearly appear that they all describe the disease, as commencing with sense of soreness, or exquisite tenderness, in the region of the the uterus, and that where it proves fatal, the appearances on dissection are those which afford the most unequivocal proofs of inflammation of the pelvic and abdominal viscera. Dr. William Hunter says 'The uterus, all the viscera, and every other part are found inflamed. There is a quantity of purulent matter in the cavity of the abdomen, and the intestines are all glued together.' The account of the morbid changes of structure by Drs. Hulme, Joseph Clarke, Gordon, Campbell, Mackintosh, and others, is nearly the same, and Dr. Hamilton, who believes that puerperal fever is a fever *sui generis*, nevertheless admits that the appearances on dissection are exactly similar to the descriptions generally given by these authors, and that acute pain of the abdomen is a primary, and not a secondary symptom of the disease. Dr. H. positively affirms, that puerperal fever is a disease of a putrid or typhoid nature, requiring for its treatment, wine, volatile alkali, bark, glysters, and animal jellies; and yet in direct opposition to his theoretical views, and as if involuntarily led by the symptoms to a correct conclusion respecting the true character of the affection, he has laid down as the first indication of treatment 'to moderate local inflammation by purging and hot fomentations.'

Dr. John Clarke admits that in most cases of the true epidemic puerperal fever, there has been some degree of inflammation in the cavity of the abdomen, and that the uterus and ovaria sometimes partake of the inflammation. In two cases which he met with there was an appearance of pus in the veins of the uterus. The brain was always in a natural state. In one instance only was there an appearance of disease in the chest. The effusion of sero-purulent fluid into the sac of the peritoneum, was so disproportioned however to the degree of inflammation, that he supposed it to arise from another cause than inflammation. It is now however admitted by all pathologists, that these copious effusions into the peritoneal sac, are invariably the result of acute inflammation of the peritoneum, and not of any peculiar disposition of the vessels of the part affected, as Dr. Clarke had supposed.

Dr. Gooch, the latest author of observations on puerperal fever in this country, has accurately described the symptoms and treatment of puerperal peritonitis. As a substitute for the ordinary names, child-bed fever, puerperal fever, and peritonitis, he has employed the term peritoneal fever 'to express the fact that an affection of the peritoneum is an essential accompaniment of the disease, without defining what that affection is, because it is not uniform.' This term peritoneal fever is perhaps the least appropriate that Dr. Gooch could have invented, for he admits that the disease may occur in its most exquisite form and yet leave few or no traces in the peritoneum after death, by which we might have been enabled to determine that this membrane had previously been the seat of the disease." 451.

Dr. Gooch remarks it as a thing very unaccountable that some of the worst forms of puerperal fever leave little or no trace of disease in the peritoneum after death. But our author has pertinently observed that Dr. Gooch appears to have been satisfied with simply inspecting the serous surface of the uterus; whereas he, Dr. Lee, is strongly inclined to believe that, had he (Dr. G.) gone behind the peritoneum, and carefully examined the spermatic and hypogastric veins, the absorbents, the uterus and its appendages, with the sub-peritoneal tissues, he would frequently have found the products of inflammation. The absence of increased vascularity in the peritoneum, and of lymph or serum in its cavity, are no proofs at all that the subjacent tissues are in a sound state. Dr. Lee very properly differs from Dr. Gooch in the reliance which is to be placed on the effects of remedies, as pointing out the nature of puerperal fever. Nothing can be more contradictory or conflicting than the testimony of authors on the subject of remedies. The study of morbid anatomy, is, assuredly, a *sine qua non* in the investigation.

"That diffused pain of the abdomen with a rapid, soft pulse, not unfrequently occurs, at particular seasons, without inflammation, or with a very slight degree of inflammation, in delicate nervous women after parturition, and that these symptoms are relieved by opiates and warm fomentations, without either general or local blood-letting, will readily be admitted. That such cases are however, if not essentially different in their nature, at least widely different in degree of severity, from cases of sporadic or epidemic puerperal fever or uterine inflammation, is clearly proved by the following observation of Dr. Gooch himself. 'There seemed to be nothing dangerous in this form of disease, provided the nature of it was not mistaken, and improper remedies not used, yet it so strikingly resembled peritoneal inflammation that it was invariably taken for it by the practitioners who witnessed it.' The results of the practice in the Westminster Lying-in Hospital in the years 1828 and 1829, still more decidedly prove that the cases described by Dr. Gooch were not cases of low child-bed fever, for of twenty-eight women who were attacked with the disease and were treated, as he had recommended, with Dover's powder, and warm cataplasms, seven died, or one in four." 455.

### Treatment of Uterine Inflammation.

This varies so much, as to severity, in different cases and in different seasons, that much modification of treatment must, from time to time, obtain. At some periods there is a marked disposition to the disease, evinced by tenderness of the uterus on pressure, and acceleration of the pulse, where inflammation is not actually developed, or where it takes place in so slight a degree as to yield to anodyne remedies and fomentations. Professor Chaussier was so convinced of the necessity of a continued and gentle perspiration to prevent and to combat puerperal peritonitis, that he made every woman recently delivered take, from time to time, and at intervals more or less distant, small doses of Dover's powder, while emollient cataplasms were applied to the abdomen.

Where peritoneal inflammation, however, is fully developed, such treatment will not avail.

"In no inflammatory disease, are the good effects of blood-letting more strikingly observed than in the first variety of uterine inflammation, puerperal peritonitis; we do not however, as Dr. Gordon has stated, possess a remedy in it which will certainly cure the disease in all cases if early applied. Where the symptoms of peritonitis manifest themselves with great violence, twenty ounces of blood should be immediately drawn from the arm, and in a few hours, if relief is not obtained, ℥xvj. more should be abstracted. The first general bleeding should be followed without loss of time by the application of leeches to the abdomen, regulating their number by the severity of the pain, and the strength of the pulse. Warm linseed meal poultices, or fomentations to the hypogastrium should invariably follow the application of the leeches; and five grains of calomel with an equal quantity of antimonial powder should be administered every two or three hours. After the second dose of this medicine, I have frequently exhibited a strong purgative draught, repeating it according to its effect. It will often be found, that the pain of the uterus continues with considerable severity after this treatment has been pursued; and that the most decided benefit results from combining half a grain or a grain of opium, or five grains of Dover's powder, with each dose of the calomel and antimony.

Where the symptoms do not indicate an attack of a formidable nature, we ought not to carry depletion so far. In a large proportion of cases, one bleeding will prove sufficient, and in many the application of leeches alone, with the internal remedies now mentioned, have subdued the disease." 457.

Oil of turpentine Dr. Lee has seen employed in a few cases without any advantage. Emetics he cannot recommend, though they have been highly praised both by French and English practitioners.

In respect to the treatment of inflammation of the uterine appendages, and the deeper-seated tissues of the uterus itself, whether of the absorbents, veins, or the muscular structure, the symptoms from the commencement, Dr. Lee thinks, do not indicate general bleeding.

"In cases where the re-action at the invasion of the disease has been violent, with acute pain of the uterus, and venesection has been employed, the relief obtained has only been temporary, if at all experienced; and, in some instances, the abstraction of only a few ounces of blood from the arm has produced syncope, or been followed by rapid sinking. Where the local pain is severe, leeches and warm fomentations seem to be the appropriate remedies; but, as far as my own observations go, we are in possession of no remedial means which effectually control those varieties of inflammation of the deeper-seated structures of the uterus which I have attempted to describe. The French physicians are, however, of a contrary opinion, and are satisfied that we possess a powerful remedy, even in the worst cases, in mercury, employed so as to excite salivation. In one case of uterine phlebitis, I pushed this remedy by inunction to a great extent, and brought the system under the influence of mercury in less than twenty-four hours; yet the progress of the symptoms was not arrested, and the patient died, as I had observed others do where the remedy had not been administered. In other cases I have employed mercury to a great extent internally, without the slightest benefit; and it may justly be doubted from the results of M. Tonellè's practice, whether or not it possesses the influence he supposes, for of forty-three cases where mercury was used as the chief remedy, only fourteen recovered." 460.

Dr. Lee, in conclusion, recommends to the serious attention of his brethren, a full inquiry as to the means of preventing the occurrence of uterine inflammation in lying-in hospitals. The records of the British Lying-in Hospital, the MATERNITÉ, the Dublin puerperal institutions, and the tables published by M. Chateau Neuf, clearly prove, that the average rate of mortality greatly exceeds that of establishments where individuals are attended at their own habitations. Should it be found that we have no effectual means of preventing this mortality, the question might fairly be raised as to the policy or humanity of keeping up such establishments at all.

We have now given a most extended analysis of Dr. Lee's paper, which is by far the most valuable in the volume of transactions under review. Dr. Lee has been eminently successful in substantiating the points of pathology which he undertakes to prove. We were not quite convinced by the facts which he brought forward, in a preceding paper, for the support of his theory of PHLEGMATIA DOLENS, and we stated our doubts. But we candidly admit that, on the present occcasion, we can scarcely start an objection to the conclusions at which this able, zealous, and unwearied physician has arrived. We trust and hope that the patronage of his brethren will encourage and foster that indefatigable spirit of investigation which Dr. Lee has for several years evinced, and successfully cultivated.

